# An analysis of the predictive factors for stone clearance at primary ureteroscopy

**DOI:** 10.1007/s11845-024-03703-8

**Published:** 2024-06-28

**Authors:** K. F. Daly, B. M Mac Curtain, E. Collins, M. Lincoln, E. MacCraith, G. Lennon, B. B. McGuire

**Affiliations:** 1 Department of Urology, St. Vincent’s University Hospital, Dublin, Ireland; 2 St. James’s Hospital, Dublin, Ireland; 3 Tallaght University Hospital, Dublin, Ireland; 4 Royal College of Surgeons in Ireland, Dublin, Ireland; 5 Royal College of Surgeons Ireland, Dublin, Ireland

**Keywords:** Ureteric colic, Ureteric stone, Ureteroscopy, Urolithiasis

## Abstract

**Background:**

Ureteric colic is a common emergency urological presentation [1]. When operative intervention is required, retrograde ureteroscopy is the most common approach. There are multiple treatment strategies including primary ureteroscopy (URS), staged ureteroscopy, and deferred ureteroscopy following ureteric stent placement. The approach is based on a number of clinical and stone factors. This study assesses the factors which predict stone clearance at the initial procedure.

**Aims:**

All patients diagnosed with an obstructing ureteric stone who were managed operatively in a consecutive 12-month period were included. Patients were evaluated for stone clearance following a single or multiple procedures. A number of factors including stone size, location, gender, age and pre-operative laboratory results were evaluated for association with stone clearance at index procedure. Multivariate logistic regression analyses were performed to produce odds ratios (OR) with confidence interval (CI) at 95% and significance values *P* < 0.05.

**Results:**

One hundred and seventy patients were included in the final analysis. Stone clearance following the index procedure was achieved in 57% (*n* = 100) of patients. Predictors of successful stone clearance at index procedure were stone size < 6 mm, male gender and distal stone location (*p* < 0.05). Proximal stone location, stone size > 10 mm and elevated c-reactive protein (CRP) were associated respectively with multiple procedures to achieve stone clearance (*p* < 0.05).

**Conclusions:**

Acute ureteric stones can be managed with a number of treatment strategies. This study identifies factors which predict stone clearance at index procedure. These results will help urologists accurately counsel patients when undertaking operative management for ureteric colic.

## Introduction

Urinary tract calculi is a common urological condition. The incidence ranges from 4 to 20% in economically developed countries [2]. The incidence of ureteric calculi presenting with colic varies based on a number of factors. It is rising in most developed countries and has been reported as high as 340 per 100,000 per year in some populations [3, 4]. This represents a large proportion of emergency urological presentations.

Management options include medical expulsion therapy, URS and extracorporeal shockwave lithotripsy (ESWL). For stones located in the ureter, medical management options include supported stone passage, while non-medical options include shock wave lithotripsy (SWL), ureteroscopy (URS) and ureterolithotomy. There are multiple strategies when undertaking endoscopic management of ureteric stones. These include primary URS, staged URS and deferred URS following ureteric stent placement. The management strategy is based on factors including surgeon preference, resource availability, and clinical and stone factors. Primary URS offers the benefit of eliminating the need for further procedures and decreasing indwelling stent time. Achieving stone clearance in a single procedure offers clear benefits in terms of resource allocation and patient quality of life [5]. However, this is not always feasible. There are multiple factors that can favour a deferred or staged approach. These include the presence of urinary tract infection (UTI), unfavorable ureteric anatomy, and surgeon or patient factors that favor treatment in an elective setting.

This study aims to evaluate the patient, clinical, and stone factors that predict clearance at primary URS versus staged procedures and primary stent placement with deferred URS. This study will aid both patients and urologists in counseling on the likely treatment outcome. It furthermore aims to identify patients who are likely to achieve stone clearance in the emergent setting.

## Methods

### Study design

We identified patients presenting with urinary tract calculus in a 12-month period January 2021 to December 2021 via hospital inpatient enquiry (HIPE) data. We identified patients with the following three diagnoses on discharge- calculus of kidney, calculus of kidney with calculus of ureter, and hydronephrosis with renal ureteric calculus obstruction. We retrospectively reviewed clinical notes and radiological imaging of all patients identified. The inclusion criteria were patients with ureteric calculi diagnosed on CT renal stone protocol, age > 18, and managed with emergency ureteroscopy during admission. Exclusion criteria were conservative management with medical expulsion therapy, renal stones identified and managed electively, and staghorn calculi.

### Measured parameters

We examined the clinical course of all patients who met the inclusion criteria. Data was collected on stone characteristics including- location (proximal, middle, or distal ureter), size (mm) measured as maximal diameter on axial, coronal, or sagittal images, number of stones(single or multiple), and the presence or absence of concurrent renal stone. We evaluated the role of demographic factors, age and gender, and clinical factors including white cell count (WCC), c-reactive protein (CRP), and serum creatinine level in predicting stone clearance.

All procedures were performed under general anesthesia. Both semi-rigid 6.5Fr and Boston Scientific LithoVue™ flexible 9.5 Fr ureteroscopes were available and the equipment used was decided by the operating surgeon. A Boston Scientific LightTrail™ Holium laser with 273 or 365-micron fibers was used for lithotripsy.

### Statistical analysis

Numerical data are presented as mean ± standard deviation. Categorical data are given as percentages. The odds ratios (OR) and 95% confidence intervals (CI) were calculated using univariate and multivariate analysis. Initially, univariate analysis was used to identify associations between covariates and the need for repeat procedures. Statistically significant results were then included in the multivariate regression analysis to determine independent risk factors. All statistical analysis was executed using IBM SPSS statistics version 28.0.

## Results

### Patient characteristics

The clinical notes were reviewed of all patients identified via HIPE data. 306 patients were identified through the initial search criteria. One hundred thirty-six patients were excluded as per the exclusion criteria and duplicates in the search findings. One hundred seventy patients were included in the study. The patient demographics are detailed in Table 1. The mean age was 50.5 ± 15.9. The mean stone size was 6.7 mm ± 3.2. The ureteral stone locations were proximal 41% (*n* = 70), mid 11% (*n* = 18), and distal 45% (*n* = 76). Data was not available in 6 patients (3%). The mean WCC, CRP, and serum creatinine were 9.8 (10^9/^L), 26.9 mg/L, and 109 mmol/L respectively.

**Table 1:** Patient Characteristics

**Variable**	**Value (%)**
**Patients**	**170**
**Mean (yrs) ± SD pt age (range)**	**50.5 ± 15.9 (16–85)**
**Mean (mm) ± SD mm initial stone diameter (range)**	**6.7 ± 3.2 (1–20)**
**Gender**	
Male	116 (68)
Female	54 (31)
**Age**	
< 50	88 (52)
> 50	82 (48)
**Stone Size**	
0–5 mm	48 (28)
6–10 mm	103 (61)
> 10 mm	19 (11)
**Location**	
Proximal	70 (41)
Middle	18 (11)
Distal	76 (45)
Missing	6 (3)
**Number of stones**	
Single	150 (88)
Multiple	20 (12)
**WCC**	
< 11	113 (66)
> 11	52 (31)
Missing	5 (3)
**Creatinine**	
< 90	68 (40)
> 90	96 (57)
Missing	6 (3)
**CRP**	
< 20	122 (72)
> 20	36 (21)
Missing	12 (7)

### Primary stone clearance rates

Stone clearance following the index procedure was achieved in 57% (*n* = 97). This was 27/70(38%) in proximal stones, 11/18(61%) mid ureteric stones and 55/76(72%) in distal stones. Clearance rates based on location were proximal 38% (*n* = 27/70), mid 61% (*n* = 11/18), and distal 72% (*n* = 55/76) (Table 2). Stone clearance was confirmed on post-operative XR or CT based on clinical factors and the preference of the treating urologist.

**Table 2:** Stone clearance at Primary Ureteroscopy based on stone location

**Stone Clearance**	**Yes**	**No**	
**Stone Location (n) (%)**			**Total**
Total	97(*57*)	73(*43*)	170
Proximal	27(*38*)	43(*62*)	70
Mid	11(*61*)	7(*39*)	18
Distal	55(*72*)	21(*28*)	76
Unknown	4(*67*)	2(*33*)	6

We evaluated factors associated with stone clearance at primary URS. Univariate analysis (Table 3) was used to identify potential predictive factors. This identified male gender (*p* = 0.01) and distal stone location (*p* < 0.001) as associated with stone clearance at index procedure (OR 0.42, 0.24 respectively). Larger stone size > 10 mm (*p* = 0.003), and elevated CRP (*p* = 0.03) were associated with the need for multiple procedures (OR 11.33, 2.31 respectively). Age, number of stones, WCC, and serum creatinine did not predict number of procedures (*P* > 0.05).Multivariate regression analysis (Table 4) was then used to identify the independent predictors for stone clearance at the initial procedure. Male gender (OR 0.44, p = 0.04) and distal stone location (OR 0.26, p = 0.001) were associated with stone clearance at the primary procedure. Elevated CRP and increasing stone size showed a trend toward predicting multiple procedures but were not statistically significant in our multivariate analysis.

**Table 3:** Univariate regression analysis

Variable	OR (95%CI)	P
**Gender**		
Female	1 (reference)	
Male	0.42 (0.22–0.81)	**0.01**
**Age**		
< 50	1 (reference)	
> 50	1.44 (0.78–2.65)	0.24
**M/S stones**		
Single	1 (reference)	
Multiple	1.38 (0.54–3.52)	0.50
**Stone Location**		** < 0.001**
Proximal	1 (reference)	
Middle	0.40 (0.138–1.16)	0.09
Distal	0.24 (0.12–0.48)	** < 0.001**
**Stone Size**		0.003
1–5 mm	1 (reference)	
6–10 mm	1.88 (0.90–3.93	0.09
> 10 mm	11.33 (2.81–45.67)	** < 0.001**
**WCC**		
< 11	1 (reference)	
> 11	1.51(0.78–2.93)	0.221
**CRP**		
< 20	1 (reference)	
> 20	2.31 (1.09–4.93)	**0.03**
**Creatinine**		
< 90	1 (reference)	
> 90	1.52 (0.80 – 2.87)	0.198

**Table 4:** Multivariate regression analysis

Variable	OR (95%CI)	P
**Gender**		**0.04**
Female	1 (reference)	
Male	0.44 (0.22 – 0.96)	
**Stone Location**		**0.04**
Proximal	1 (reference)	
Middle	0.59 (0.19 – 1.86)	0.37
Distal	0.26 (0.12 – 0.58)	**0.001**
**Stone Size**		0.30
1–5 mm	1 (reference)	
6–10 mm	1.36 (0.58 – 3.18)	0.48
> 10 mm	3.38 (0.72–15.88)	0.12
**CRP**		
< 20	1 (reference)	
> 20	1.90 (0.80 – 4.48)	0.14

## Discussion

This study has identified predictors of positive and negative predictors of successful primary URS in the treatment of urolithiasis. Positive predictors are male gender, stone size < 6 mm, and distal ureteral location. Negative predictors are elevated CRP, stone size > 10 mm, and proximal ureteral location. This study provides urologists with useful information in terms of operative planning and patient counseling.

There are a small number of studies assessing clinical stone and stone factors that predict stone clearance at primary ureteroscopy. The Clinical Research office of Endourological Society (CROES) Global study reports on the overall pre-stenting rate of 11.9% in patients with ureteric stones [6]. However, there was significant variability with a > 50% rate in Germany and a 37.8% in patients undergoing flexible URS for renal stones [6]. Their study found increasing ASA scores and increased BMI predicted pre-stenting. Larger stone size was associated with a lower rate of pre-stenting which is in contrast to our study. However, it is noted that their data collection did not allow for the capture of patients who underwent multiple URS to achieve stone clearance as each procedure was captured as a new patient.

While it is demonstrated in the CROES Global study that overall rates of pre-stenting are low, there are centers that utilize a pre-URS stent regularly. Lumma et al. [7] describe their series of 550 ureteroscopies with an 88.4% rate of prior stent placement. They found that pre-stenting improved SFRs and decreased complication rates. This improvement in SFR was greatest in mid and proximal ureteric stones(67.1 vs 34.5%). This represents an almost uniform policy of pre-stenting. The increased rate of stone clearance and decreased complication rate must be weighed against the economic cost of more procedures and significant morbidity associated with ureteric stents [8].

Further studies have been less compelling in regard to the benefit of pre-stenting. Navetta et al. [9] in their study of 421 ureteroscopies did not find an increased SFR in the pre-stented cohort. Similarly, Shields et al. [10] did not find pre-stenting increased stone clearance at ureteroscopy.

This compares to 43% of patients who had pre-stenting or multiple URS to achieve stone clearance. Our study aims to identify factors that are associated with stone clearance at primary ureteroscopy. There is limited evidence in this area. One of the only studies by Tran et al. [11] uses a novel scoring system incorporating change in serum creatinine level and peri-ureteral density on CT scans to stratify patients’ risk of stone clearance. Their study of 247 patients had a total SFR of (81.8%). Their study found distal stone location was associated with stone clearance as was elevated serum creatinine and increased trend-ureteral density measured on CT. In contrast to our findings, they did not find a difference in SFRs based on gender. It is unclear why the male gender appears to predict stone clearance at primary URS however we postulate it may be due to anatomical differences in ureteric anatomy between males and females. Elevated CRP was associated with a trend towards multiple procedures, we believe it is because it represents concomitant urinary infection influencing management in favor of primary stent insertion and interval URS.

Our patients represent a heterogeneous group and the rationale for a repeat procedure is varied. A limitation of our study is the reason for repeat procedure is not captured in the data. A further limitation is its retrospective nature. However, our data represents real-world practice with the inclusion of all acute URS performed in a 12-month period.

## Conclusion

In this study, we identified predictors of successful primary URS in the management of ureteral calculi. The primary URS success rate is 57%. Positive predictors are male gender, stone size < 6 mm, and distal ureteral location. Negative predictors are elevated CRP, stone size > 10 mm, and proximal ureteral location. This study provides urologists with useful information in terms of operative planning and patient counseling.

## Abbreviations

URS: Ureteroscopy
ESWL: Extracorporeal shockwave lithotripsy
UTI: Urinary tract infection
XR: X-ray
CT: Computerized topography

## References

[1] Daudon M, Haymann J-P, Estrade V et al (2023) 2022 Recommendations of the AFU Lithiasis Committee: Epidemiology, stone analysis and composition. Prog en Urol J l’Association Fr d’urologie la Soc Fr d’urologie 33(14):737–765 10.1016/j.purol.2023.08.013 37918977

[2] Trinchieri A (2008) Epidemiology of urolithiasis: an update. Clin Cases Miner Bone Metab [Internet] 5(2):101–6. Available from: https://pubmed.ncbi.nlm.nih.gov/22460989 PMC2781200 22460989

[3] Loeff S, Saluja M, Rice M (2018) Review of acute symptomatic urolithiasis in Auckland. N Z Med J 131(1469):44–50 29389928

[4] Fwu C-W, Eggers PW, Kimmel PL, Kusek JW, Kirkali Z (2013) Emergency department visits, use of imaging, and drugs for urolithiasis have increased in the United States. Kidney Int [Internet] 83(3):479–86. Available from: https://pubmed.ncbi.nlm.nih.gov/23283137 10.1038/ki.2012.419 PMC3587650 23283137

[5] Zargar-Shoshtari K, Anderson W, Rice M (2015) Role of emergency ureteroscopy in the management of ureteric stones: analysis of 394 cases. BJU Int [Internet] 115(6):946–50. 10.1111/bju.12841 24925167 10.1111/bju.12841

[6] Assimos D, Crisci A, Culkin D et al (2016) Preoperative JJ stent placement in ureteric and renal stone treatment: results from the Clinical Research Office of Endourological Society (CROES) ureteroscopy (URS) Global Study. BJU Int 117(4):648–654 26237735 10.1111/bju.13250

[7] Lumma PP, Schneider P, Strauss A, Plothe KD, Thelen P, Ringert RH et al (2013) Impact of ureteral stenting prior to ureterorenoscopy on stone-free rates and complications. World J Urol [Internet] 31(4):855–9. Available from: https://pubmed.ncbi.nlm.nih.gov/22037634 10.1007/s00345-011-0789-6 PMC3732763 22037634

[8] Scarneciu I, Lupu S, Pricop C, Scarneciu C (2015) Morbidity and impact on quality of life in patients with indwelling ureteral stents: a 10-year clinical experience. Pakistan J Med Sci [Internet] 31(3):522–6. Available from: https://pubmed.ncbi.nlm.nih.gov/26150836 10.12669/pjms.313.6759 PMC4485263 26150836

[9] Navetta AF, Elmekresh A, Doersch K, Durdin TD, Machen GL, Cohen A et al (2019) Preoperative ureteral stenting prior to ureteroscopy for management of urolithiasis does not impact the postoperative return for unplanned care. Urol Ann [Internet] 11(3):282–6. Available from: https://pubmed.ncbi.nlm.nih.gov/31413507 10.4103/UA.UA_78_18 PMC6676851 31413507

[10] Sj M, Bv G, Reid G, Orlando G-M (2009) Impact of preoperative ureteral stenting on outcome of ureteroscopic treatment for urinary lithiasis. J Urol [Internet] 182(6):2768–74. 10.1016/j.juro.2009.08.043 19837426 10.1016/j.juro.2009.08.043

[11] Tran TY, Hernandez Bustos N, Kambadakone A et al (2017) Emergency ureteral stone treatment score predicts outcomes of ureteroscopic intervention in acute obstructive uropathy secondary to urolithiasis. J Endourol 31(9):829–834 28637368 10.1089/end.2017.0043

